# Validation framework for *in vivo* digital measures

**DOI:** 10.3389/ftox.2024.1484895

**Published:** 2025-01-08

**Authors:** Szczepan W. Baran, Susan E. Bolin, Stefano Gaburro, Marcel M. van Gaalen, Megan R. LaFollette, Chang-Ning Liu, Sean Maguire, Lucas P. J. J. Noldus, Natalie Bratcher-Petersen, Brian R. Berridge

**Affiliations:** ^1^ VeriSIM Life, San Francisco, CA, United States; ^2^ AbbVie Inc., Chicago, IL, United States; ^3^ Tecniplast SpA, Buguggiate, Italy; ^4^ Evotec, Goettingen, Germany; ^5^ The 3Rs Collaborative, Denver, CO, United States; ^6^ Pfizer, Groton, CT, United States; ^7^ GSK, Collegeville, PA, United States; ^8^ Noldus Information Technology BV, Wageningen, Netherlands; ^9^ Radboud University, Nijmegen, Netherlands; ^10^ Digital In Vivo Alliance (DIVA), Redwood City, CA, United States

**Keywords:** digital biomarkers, 3Rs (replace reduce refine), preclinical, translation, drug discovery and development, rodents, validation, verification

## Abstract

The adoption of *in vivo* digital measures in pharmaceutical research and development (R&D) presents an opportunity to enhance the efficiency and effectiveness of discovering and developing new therapeutics. For clinical measures, the Digital Medicine Society’s (DiMe) V3 Framework is a comprehensive validation framework that encompasses verification, analytical validation, and clinical validation. This manuscript describes collaborative efforts to adapt this framework to ensure the reliability and relevance of digital measures for a preclinical context. Verification ensures that digital technologies accurately capture and store raw data. Analytical validation assesses the precision and accuracy of algorithms that transform raw data into meaningful biological metrics. Clinical validation confirms that these digital measures accurately reflect the biological or functional states in animal models relevant to their context of use. By widely adopting this structured approach, stakeholders—including researchers, technology developers, and regulators—can enhance the reliability and applicability of digital measures in preclinical research, ultimately supporting more robust and translatable drug discovery and development processes.

## 1 Introduction

*In vivo* digital measures are quantitative measures of biological events or processes using digital technologies applied directly to an animal or incorporated into an animal’s cage that could be considered analogous to digital health technologies used in clinical trials. Applying *in vivo* digital measures in drug discovery and development is an important addition to the growing portfolio of novel tools that can improve the efficiency and effectiveness of efforts to discover and develop new medicines (Baran et al., 2022). However, as with other novel tools, their near- and long-term impact depends on developing the confidence that these measures provide meaningful information about the biology of the animals used to model the human patient. To achieve this confidence, “validation” is the evidence-building process often applied to quantitative assays and their outputs to support their analytical performance and ‘clinical’ relevance, respectively.

To bolster discussions and common understanding, we have clarified the terminology used in this manuscript in Table 1. While alternate definitions and terms may have equal merit, these definitions were chosen to communicate the concepts and principles in a simple but flexible manner that aligns with the terminology used in the clinical space. Although the term “digital biomarker” has been commonly used in the past (Baran et al., 2022), based on recent discussions within our groups and in alignment with recent publications (Izmailova et al., 2023; Macias Alonso et al., 2024; Leyens et al., 2024) we now adopt the term ‘digital measure’ going forward, as we believe it better aligns with the context (See Table 1). For this manuscript, we do not reference terminology related to digital measures of micro- and macro-environmental factors, as while these measures are significant, they are not the focus of this paper.

**TABLE 1:** Terms, definitions, and related references for verification of digital biomarkers.

Term	Definition	References
Clinical	Studies that involve the treatment and/or monitoring of living human patients	
Preclinical and Nonclinical	The terms preclinical and nonclinical are often used interchangeably to refer to studies utilizing in vivo, in vitro and in silico methods as part of the research and development process. They do not involve human participants. These studies are designed to characterize the pharmacology, efficacy, safety, and disposition (e.g., absorption, distribution, metabolism, and excretion) of drug candidates to inform their clinical progression. Typically, preclinical is used to refer to in vivo studies and is therefore the preferred term in this manuscript	
Digital Health Technologies	A clinical term that is defined as electronic tools, systems, devices, and resources that generate, store, and process data in healthcare and include mobile health, wearable devices, telehealth, telemedicine, electronic health records, and patient portals	
Clinical Digital Biomarker	Characteristic or set of characteristics, collected from digital health technologies, that is measured as an indicator of normal biological processes, pathogenic processes, or responses to an exposure or intervention, including therapeutic interventions	Digital biomarkers: Convergence of digital health technologies and biomarkers (nature.com)
Digital In Vivo Technologies	Digital in vivo technologies include both internal (e.g., injectable, ingestible, surgically implanted) and external (e.g., wearable, camera, microphone, electromagnetic field detector) sensors that are used to collect data from research animals. This can also refer to the systems that process this data	
Digital measure (in vivo)	Digital measures, as related to in vivo research, are quantitative data collected continuously from unrestrained and uninstrumented animals using digital in vivo technologies	
Digital Biomarker	A digital biomarker is an objective, quantifiable digital measure of physiological and/or behavioral response to disease progression or therapeutic intervention. They can be characterized as exploratory, validated, or qualified based on their maturity in a specific context of use	Definition adopted and updated from https://www.ncbi.nlm.nih.gov/pmc/articles/PMC8885444/
Translational Digital Biomarker	A translational digital biomarker is a digital biomarker that has been determined to be clinically relevant and translates between preclinical and clinical studies	
Home cage or home environment	The cages and environments where animals are housed for the majority of their lifetime in the vivarium	https://www.ncbi.nlm.nih.gov/pmc/articles/PMC8885444/
Clinical validation	Demonstrating that technology adequately identifies, measures, or predicts a meaningful clinical, biological, physical, functional state or experience in the specified (Baran et al., 2022) animal cohort and (Izmailova et al., 2023) context of use	https://www.ncbi.nlm.nih.gov/pmc/articles/PMC8885444/#B20
Benchmarking	Rigorous comparison of the performance of a novel method with a more established method to demonstrate equivalent or better performance and value	Nonhuman Primate Model Systems State of the Science and Future Needs | National Academies
Context of use	The manner and purpose of use for a technology or approach (how and when it will be used). This term can generally be applied to any intended use of a methodology. Contexts of use (COU) elements include what is measured and in what form, and the purpose of the technology or approach in the testing of hypotheses or decision-making/action	https://pubmed.ncbi.nlm.nih.gov/35064273/ https://www.fda.gov/media/109634/download https://www.fda.gov/media/133511/download Catalyzing the Development and Use of Novel Alternative Methods (nih.gov)

The digital measure process begins with the collection of a raw signal by digital sensors which are transformed into quantitative measures of behavioral and/or physiological function, and, finally, transferred into a software platform for analysis, reporting, and visualization. Each of these components of digital measure development should be assessed in some way to support the validity of the final digital quantitative output. For this paper, we defer to manufacturers to apply industry standards for validating the performance of the sensor technologies (e.g., digital video cameras, photobeam, electromagnetic fields (EMF), radio-frequency identification (RFID) readers, biosensors, microelectronics, etc.), associated firmware and supporting data acquisition software. Our focus is validating data processing software (non-AI and AI) algorithms and their quantitative outputs. The algorithms can be considered novel “assays” to which the usual principles of analytical validation apply.

The Digital Medicine Society (DiMe) recently published the V3 (Verification, Analytical Validation, and Clinical Validation) Framework as a construct within which to build a body of evidence that supports the reliability and relevance of Biometric Monitoring Technologies (BioMeTs) applied in a clinical setting (Goldsack et al., 2020). DiMe is an independent organization dedicated to delivering clinical-quality digital medicine resources on a tech timeline. BioMeTs are defined by DiMe as “…connected digital medicine products that process data captured by mobile technologies using algorithms to generate measures of behavioral and/or physiological function”. This framework distinguishes the verification of source data and the device that generated it from the analytical validation of the algorithm, and from the clinical validation of the biological relevance of the quantitative output within its intended context of use. This framework was derived from existing regulatory guidelines and represented as analogous to the FDA’s Bioanalytical Method Validation Guidance for Industry (Goldsack et al., 2020; FDA, 2020).

The 3Rs Collaborative’s (3RsC) Translational Digital Biomarkers (TDB) initiative recently published a review of the emerging role of digital measures derived from animal home cage monitoring in which they referenced DiMe efforts and briefly discussed technology verification, analytical validation, and clinical validation (Baran et al., 2022). The 3RsC is an independent non-profit organization whose mission is to advance better science–for both people and animals–through the 3Rs of animal research: replacement, reduction and refinement. It facilitates collaborative efforts across all stakeholders including academia, industry, and more. One such effort is focused on increasing industry adoption and regulatory acceptance of TDB by bringing together a broad stakeholder group of developers and end-users.

In this publication, we are building on these initial efforts by providing more detailed guidance on the use of an “*In Vivo* V3 Framework” as an adaptation of the V3 Framework first described by Goldsack, et al. This adaptation was first proposed by the Digital *In Vivo* Alliance (DIVA), a precompetitive and multi-disciplinary collaboration of preclinical scientists and data scientists, as a key element of its digital biomarker discovery and development paradigm (DIVA, 2024).

Adopting this clinical framework for preclinical use is useful for several reasons. The holistic scope of the framework ensures addressing key sources of data integrity throughout its life cycle from its raw source (i.e., verification) to its transformation into quantitative measures (i.e., analytical validation) and application in biological interpretation and translational decision-making (i.e., clinical validation). Also, replicating this clinical approach to building confidence in these novel measures strengthens the line of sight between these two distinct but interdependent drug development efforts. Additionally, it enables us to fully apply the learnings from the growing experience in validating clinical measures for clinical decision-making.

Unlike the DiMe V3 Framework, the *in vivo* V3 Framework is specifically tailored to address the unique requirements and variability of preclinical animal models. It incorporates validation strategies that are adapted to the complexity of animal models, ensuring that digital tools are validated not only for their analytical performance but also for their biological relevance within a preclinical context. The key distinction between the DiMe V3 Framework and the *in vivo* V3 Framework lies in their respective scopes. The *in vivo* V3 Framework must account for challenges unique to preclinical research, such as the need for sensor verification in variable environments, and analytical validation that ensures data outputs accurately reflect intended physiological or behavioral constructs. This adaptation is crucial in preclinical research, where the goal is to establish translational relevance and scientific insights rather than immediate clinical utility. The framework also emphasizes replicability across species and experimental setups—an aspect that is critical due to the inherent variability in animal models.

The responsibility for applying this *in vivo* framework is shared by developers, vendors, and end users, depending on technologies, maturity of the measure, study use, and scale. This paper supports this adaptation by communicating the underlying concepts and principles behind validation as applied to digital measures in in vivo animal studies.

**FIGURE 1:**
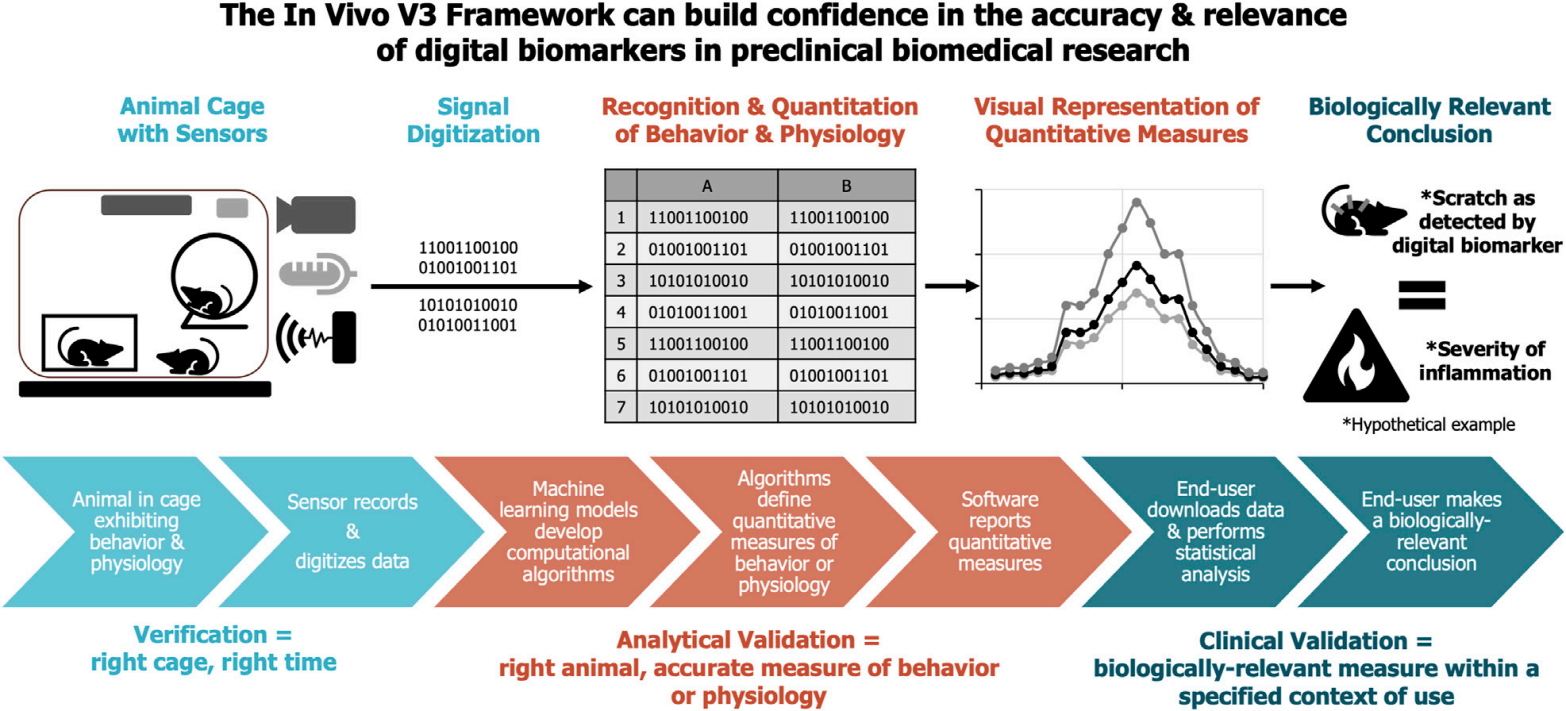
*In vivo* V3 Framework. this framework builds confidence in the accuracy and relevance of digital biomarkers in preclinical biomedical research. The figure visually represents the 3 stages of verification, analytical validation, and clinical validation including the data supply chain framework. This figure is adapted and expanded from Berridge et al. (2023).

For clinical measures, Goldsack, et al. (2020) proposed a “data supply chain” model, which serves as a beneficial guide for understanding where verification and validation are applied (see Figure 1). This model is also useful when considering digital measures within *in vivo* animal studies. In these studies, digital technology is attached to or implanted in the animal or incorporated into the home cage or a test environment for a specific assessment. Digital sensors collect and digitize raw data, which is then organized and stored either locally or remotely, along with its corresponding metadata identifiers. Algorithms are used to convert this raw data into quantifiable representations of the recorded behavioral or physiological events, such as activity, locomotion, or respiration. These algorithms and/or technology’s resolution determine the temporal resolution of the data (e.g., centimeters traveled per second, percentage of time spent in locomotion, breaths per minute, and core body temperature at minute(s) intervals. This concept also applies to spatial parameters, especially relevant for behavioral measures (time spent in a zone, distance between animals, etc.), as they depend on the spatial resolution of the technology (e.g., a video camera has a higher resolution than a matrix of photobeams depending on the density of the beams). The transformed, quantitative data is then exported for statistical analysis or visualization, typically supported by a software platform. The results of this statistical analysis of the data provide insights for study interpretations and guide subsequent development decisions.

## 2 Metadata, raw data, and consensus data

In animal research, particularly in the context of validating digital measures, accurate metadata, raw data, and consensus data are important.

Metadata, a term sometimes referred to as “data about data,” plays a vital role in providing both essential context for study findings and critical information needed to reproduce a study (Moresis et al., 2024). Metadata plays a crucial role in providing context to raw data by providing information on the circumstances in which the data were gathered. Metadata includes two types of information: the study protocol metadata (which describes the conditions that apply to all study subjects and experimental trials) and the independent variables metadata (which often vary between groups within one experiment but are generally constant for the duration of a trial). The latter include variables generated by the system (date, time, trial duration, etc.) and user-defined independent variables (e.g., animal ID, treatment, dosage). Metadata serves as a navigational tool, providing guidance for interpretations and analyses, and guaranteeing that the data can be comprehended and utilized effectively by fellow researchers. Including such a comprehensive degree of detail is of utmost importance in ensuring the study’s interpretability and integrity by providing thorough information, it also enables other researchers to replicate the experiment under comparable circumstances, thereby verifying the results obtained.

In contrast, raw data refers to unmodified outcomes derived from observations or measures which is generally the primary analytical substrate of interest to the investigator. Raw data play a fundamental role in scientific investigation since they constitute an unprocessed account of experimental observations that are crucial for the verification and reproduction of research results. The careful acquisition, retention, and dissemination of unprocessed data are important as they allow scholars to track the path of their research, examine the technique employed, and independently validate the findings. Digital raw data should be collected and stored in adherence with “FAIR” principles (i.e., findable, accessible, interoperable, reusable) of data management (Wilkinson et al., 2016).

The utilization of consensus data is of importance in guaranteeing uniformity and synchronization within research studies. Researchers can ensure the consistency, comparability, and interoperability of data across various studies and platforms by attaining a consensus on data standards and procedures. This is particularly crucial in collaborative research settings where numerous teams or institutions may be engaged in interconnected initiatives. The use of a consensus strategy in research endeavors facilitates a shared understanding among participants, consequently augmenting the credibility and dependability of research findings. The inclusion of consensus data in study findings enhances their credibility by demonstrating conformity to established standards and best practices.

Combining raw data, metadata, and consensus data is crucial in information analysis. Raw data serves as the fundamental source of information, while metadata serves as a framework that enables effective interpretation of this information. Additionally, consensus data ensures the establishment of standardized and credible information. The careful handling and organization of these data categories are crucial for maintaining the accuracy and replicability of study outcomes. Additionally, raw data and applicable metadata allow for its repurposing where novel digital measures can be extracted without running new studies (Moresis et al., 2024; Fuochi et al., 2023).

## 3 *In vivo* V3 Framework for preclinical applications

### 3.1 Protocols

Verification and validation are processes that are applied to sensor-derived digital data and the algorithms that transform those data into a useable analytical form to ensure their accuracy and relevance for the decision being made. Essential aspects of the verification and validation process should be pre-defined by comprehensive protocols that specify the endpoint(s) for validation and delineate the actions and the anticipated outcomes for each segment of the verification and validation process. These protocols should assign responsibility for the conduct of the verification or validation and establish the time of application (e.g., when during the development or life cycle of a digital measure), the method to be employed, and the relevant context of use. The results derived from these protocols then serve as valuable resources for end-users of these measures and should be included with commercial products and referenced in scientific publications that present these *in vivo* digital measures.

### 3.2 Verification

This step assumes that technology verification, which ensures that the technology, including hardware and software components, functions correctly and reliably, has already been completed by the developer or manufacturer of that technology.

Verification is the process of confirming the source and correct identification of primary sensor data, ensuring the integrity of the raw data used as input for appropriate algorithms. These algorithms transform the raw data into quantitative metrics that represent biological events from the specific cage it was collected. For instance, for computer vision sensors, verification includes ensuring proper illumination (intensity level), contrast between animal and background or bedding, visibility of a marker (if appropriate), absence of reflection (e.g., in a water maze), that the camera is recording events from the correct cage, with the right animals, for the designated study, at the correct timestamp. Similar checks are applied to other technologies like photo beam breaks, capacitance, or RFID to ensure data is collected from the intended animals and matches the metadata identifying the source.

This verification process (Figure 1) is a key quality assurance step involving digital measures, akin to standard quality assurance (QA) processes in drug development, usually conducted primarily by technology providers and may be confirmed by end-users. Furthermore, additional QA checks should be performed at the end-user level. For instance, a protocol, which could be a checklist, guides the verification by documenting sensor function, correct data labeling, and proper data storage, ensuring data integrity and continuity from study initiation to termination. This includes checks at study completion to confirm that data collection covered the intended period, identifiers remained consistent, and the data was preserved without corruption.

### 3.3 Analytical validation

Analytical validation ensures that the quantitative metrics reported by an algorithm accurately represent what the digital measure captured in the cage at the time that the technology time stamp said it did, as an estimate of what truly happened in the cage, and at the quantitative resolution intended. Analytical validation is an affirmation of data accuracy and precision, as well as its level of sensitivity and specificity irrespective of context of use or *post hoc* analyses. Alternatively, the specific context of use will define the acceptable limits of these attributes and whether those limits meet expectations for clinical usefulness.

The approach to analytical validation depends on the biological, behavioral, or physiological function being detected and measured. It requires a clear definition of that function which should be established at the start of the algorithm development process. This becomes more challenging with unsupervised algorithm development, where the algorithm may identify a biological feature that we can’t define or visualize. The ability to prospectively define the function of interest (i.e., that is being measured) is a key distinction between supervised and unsupervised digital measure algorithm development. Unsupervised approaches offer the opportunity for useful discovery of novel endpoints or biomarkers but may be more difficult to validate.

Analytical validation can be the most challenging component of the V3 Framework since digital technologies often measure biological events at resolutions greater than humans can detect and may be of higher temporal resolution, greater precision and/or accuracy than a “gold standard” or comparator measure (Van Dam et al., 2023). Also, there may not be an existing comparator to apply in the validation process (i.e., when measuring a unique measure). Accordingly, a weight of evidence or ‘triangulation’ process may be necessary using biological plausibility, comparison to an existing analytical reference standard or reasonable surrogate, and direct observation and annotation for those technology outputs that are amenable to direct observation (e.g., computer vision). The collective assessment of each of these features (measurable properties or characteristics used by an algorithm to make predictions or understand patterns) provides a level of confidence greater than any one by itself, particularly when no single one of them is a perfect standard.

The assessment of features can be done using interpretability tools (methods used to understand how an algorithm makes its decisions) such as SHAP (SHapley Additive exPlanations), LIME (Local Interpretable Model-Agnostic Explanations), Grad-CAM (Gradient-weighted Class Activation Mapping), and permutation feature importance, each serving a unique purpose. SHAP values provide an understanding of how much each feature contributes to a model’s output, helping to discern if the features being used align with biological expectations. LIME, on the other hand, allows a local approximation of the model behavior, giving insights into how specific features impact individual predictions, which can be helpful in validating the algorithm’s behavior in different contexts of use.

For deep learning models, particularly those utilizing convolutional neural networks, tools like Grad-CAM help visualize which parts of an image or input are contributing most to the prediction. This is crucial when attempting to understand how well the algorithm “sees” and processes data, ensuring that it focuses on meaningful biological signals rather than background noise or artifacts. For example, in a home-cage monitoring scenario, Grad-CAM could verify whether an algorithm assessing activity is truly focusing on the movement of the animal rather than irrelevant elements such as cage bedding.

Moreover, permutation feature importance can be applied to estimate the drop in model performance when the values of a particular feature are shuffled. This method helps identify which features are truly significant for output, indicating their biological relevance. By understanding feature importance, researchers can also determine if the model is utilizing spurious correlations that do not make biological sense, thus preventing overfitting the model to artifacts that might be specific to a certain dataset or environment.

Integrating these interpretability assessments into the validation framework provides transparency in understanding how the algorithm arrives at its output. By applying tools like SHAP, LIME, and Grad-CAM within the framework, researchers can gain insights into feature importance, validate model behavior in specific contexts of use, and ensure that the model’s focus aligns with biologically relevant signals. For example, SHAP values can help identify the contribution of features such as light cycle or environmental enrichment, while Grad-CAM can visualize which parts of an image are driving predictions of behaviors like locomotion.

Interpretability assessments within the validation framework allow researchers and regulators to build confidence that digital measures are accurate reflections of biological phenomena rather than coincidental associations. Ultimately, such assessments reinforce the reliability of digital measures, aiding in their adoption for decision-making in preclinical and translational research.

While we have discussed certain methods above, it is important to note that numerous other models and tools for feature assessment and model interpretation exist beyond the scope of this work. These tools offer alternative insights and approaches that complement those we have covered. For readers interested in a broader exploration of these methods, we recommend consulting the following references (van Zyl et al., 2024; Li et al., 2022; Allgaier et al., 2023; Ali et al., 2023; Alzubaidi et al., 2021; Hosain et al., 2024).

For biological events with accepted normal ranges, algorithm-defined measures within expected normal and/or disease-associated responses provide some confidence in their legitimacy. Examples include circadian rhythm variations in rodents’ activity, locomotor velocity and distance traveled, and respiratory rates. Furthermore, analytical validation of an automated system depends on the appropriate filtering of the raw data, e.g., to separate locomotion from body motion, and track smoothing. Inappropriate filtering will lead to over- or under-estimation of the distance traveled. Technically this is easy, but it needs to be agreed on what locomotion is (e.g., spatial movement over some X distance) and what it is not (e.g., rearing, resting, sniffing, etc.). The traditional approach in the field is to first agree on the definition of the behavioral measure, then develop an automated measure system, and finally validate that system against the agreed definition. More recently, AI approaches are sometimes being used where the system starts without *a priori* defined classes and does the clustering itself (i.e., unsupervised). However, this approach is still in its infancy and not yet standard in commercial systems.

A common validation approach is to compare a measure collected in a novel way to one collected using usual approaches. Examples include comparing respiratory rates obtained with computer vision with a measure collected by plethysmography, temperature readings from an implanted RFID chip with readings collected with a manually inserted thermometer, and a digital measure of seizure derived from invasive EEG recording. For example, the breathing rate in mice as determined by a computer vision-equipped “smart cage” was validated by placing mice in a plethysmography chamber within the smart cage to collect that data simultaneously (Supplementary Material to (Baran et al., 2020)). Similarly, Tse, et al. compared digital assessments of individual animal locomotion using a computer vision-equipped home cage system to visual activity assessments included in a manual modified Irwin test (Tse et al., 2018). Though these comparisons must often account for the impact of environmental factors on the measures, they are often found to be very similar.

Understanding if these environmental factor differences could plausibly lead to differences in the examples of respiratory rate and temperature is critical in validation and supports the principle that observing the variance in response to a stimulus or condition like a test compound or a disease state may be more insightful than the absolute values of the measures themselves. For instance, while the basal respiratory rate or body temperature of a mouse might exhibit discrepancies between the digital measure reported value and the more traditionally acquired value, the response to a known respiratory rate or temperature modulator should be consistent. For example, Lynch, et al. compared the responses of rats to varying doses of chlorpromazine and caffeine using an infrared beam-based activity meter, a mechanical vibration-based system, and the traditional modified Irwin test (Lynch et al., 2011). Though the absolute measures varied across those assessments, they revealed responses (e.g., increased or decreased activity) consistent with each other and the expected pharmacology of the test articles. These types of assessments would also support clinical validation since they represent “clinical” contexts in which those measures would be applied.

The VAME framework presented by Luxem et al. (2022) focuses on unsupervised learning of behavioral motifs and hierarchical structure in animal motion (Luxem et al., 2022). The study used a Hidden Markov Model (HMM) to cluster behavioral motifs and compare these to human observations. One notable example was the identification of subtle behavioral differences in Alzheimer transgenic mice that were not detectable by human observation. This emphasizes the potential of automated digital measures to detect effects consistent with traditional methods (e.g., significant behavioral changes), even when specific measurement values may differ. This approach to developing algorithms for digital measures also involves a validation process. Specifically, the validation of these digital measures can be done by comparing the outputs of the Hidden Markov Model (HMM) to manual human observations, demonstrating consistency in identifying meaningful behavioral patterns. Additionally, the study exemplifies analytical validation by showing that the automated system can detect subtle differences beyond the resolution of human observation, thus providing evidence that the algorithm’s quantitative outputs accurately reflect biological events.

The study by Van Dam et al. (2013) used an automated behavior recognition (ABR) system to identify rat behaviors, which were then compared to manual scoring by expert annotators (van Dam et al., 2013). The validation involved measuring the behavioral effects of drug treatments and comparing automated outputs to those from manual scoring, which showed similar behavioral effects despite some discrepancies in individual measures. This practical approach shows how discrepancies were handled by verifying effect consistency through pharmacological validation, ultimately aligning the outcomes from both automated and traditional methods.

A major impetus for developing automated digital measures is to collect biological data more continuously, more sensitively, and with fewer artifacts than traditional measures. Accordingly, digital measures may be more sensitive than traditional measures undermining our use of traditional measures as a “standard” (and thus the use of a triangulation approach). In cases where the raw data can be manually reviewed (e.g., raw video), a manual validation of what the sensor “saw” and what the algorithm reported may be useful. Modern cloud technology can facilitate this approach by enabling the development of software platforms through which researchers can share annotated animal behavior and physiology data (e.g., data collected from wildtype or control animals) thus preventing intellectual property issues. By doing so, they can contribute to the development and improvement of AI models for automated collection and analysis of digital measure data, from which the entire field will benefit.

Examples could include reviewing a segment of home cage video to correlate with algorithm-reported measures such as activity, distance traveled, or velocity of that spatial movement as described in Tse, et al., 2018 where activity assessments were done using raw video as a comparison to computer vision-derived measures generated at the same time (Tse et al., 2018). Algorithm reported seizure events or time spent at a feeder could be confirmed by video review. Corrective annotations by the human observer can be used to improve the AI-based behavior recognizer (a technique called active learning). By doing so, the power of computer vision and that of the human brain are combined to increase the performance of the automated tool (van Dam et al., 2024). This approach may be more difficult when the raw data is less accessible, such as when observation is obscured, say due to nesting material or assessing non-visual events.

Analytical validation necessitates determining the amount of data to be validated to provide confidence in the data not directly validated. This can be achieved by understanding the principles and power of statistical analysis and data validation.

The frequency of the events being measured greatly influences the amount of data to be validated. If the event is rare (such as seizures), a larger sample size may be needed to ensure that enough instances of the event are captured to accurately assess true and false positives and negatives. Conversely, if the event is common (such as locomotion), a smaller sample size may suffice.

The likelihood of “false negatives” becomes a major determinant in this context because if a significant number of false negatives are present, it could lead to an underestimation of the event’s frequency or severity. Therefore, a sufficient amount of “negative” data must be reviewed to ensure its true negativity. For example, in the case of measuring behavioral “activity” in rodents during the dark cycle, if the frequency of activity is high, a smaller sample size may be sufficient. However, if the activity is infrequent or unpredictable, like seizures in an epilepsy model, a larger sample size may be needed to ensure accurate detection of both true and false positives and negatives.

The constancy and consistency of the event, such as respiration, can affect the amount of data reviewed. When detection and quantification of respiration are constant and consistent, fewer data points may be needed to establish a reliable baseline. However, if respiration is variable due to environmental or behavioral factors, more data may be needed to accurately capture this variability. The state of the animal (sleeping or locomoting) or the light cycle (light or dark) can affect the accuracy and detectability of respiration measures. For instance, an animal’s respiration rate may change depending on whether it is sleeping or active, and these changes may be detectable depending on the light conditions. When using computer vision, these factors can influence the accuracy of the measures, so they must be considered when determining the amount of data to be validated.

The sample size for analytical validation should account for bioassay performance metrics like sensitivity, specificity, accuracy, and precision. An analytical validation should also provide insights into the limits of detection/quantitation, dynamic range, and reproducibility. Statistical approaches can guide the number of “events” or “bouts” to be validated by determining the sample size needed to confidently represent the entire data set. For example, validating an algorithm’s ability to detect a bout or period of horizontal locomotion (i.e., the animal is moving across the cage floor) might involve determining how often those events might be expected to occur either from manual observation or other technology-derived data and defining a target level/or expected level of sensitivity (for example, I want to be able to identify 90% of an animal’s bouts of horizontal locomotion). Power analysis will allow us to determine how many bouts of horizontal locomotion we need to evaluate to provide confidence that an algorithm is detecting them 90% of the time. Likewise, assessing how many events the algorithm detected are horizontal locomotion *versus* some other behavior (e.g., active but not locomoting) will provide insights into the algorithm’s specificity. Modern video tracking systems can assist with addressing this challenge through their filtering functions. This aspect becomes a more significant challenge when we are trying to identify rare behaviors (for which it may be hard to collect enough training material), behaviors of which the execution by the animal is heavily impacted by the treatment (so a tool trained for wildtype mice or untreated rats is unapplicable), and in particular social behaviors (because most multi-animal video tracking systems are still not able to maintain animal identity for more than a few minutes).

Data sets used for validation should be distinct from the “training sets” used to develop the algorithm even if from the same study but, most usefully, should be from a bespoke study-i.e., a validation study (which might also serve as a “clinical” validation study). Ideally, analytical validation should be assessed on data from more than one study.

Accuracy and precision relate to the quantitative output of the algorithm. Using the previous paragraph’s example, the temporal duration of a bout of locomotory activity or distance traveled reported by an algorithm should be validated for accuracy-i.e., that the quantity reported is what happened in the cage. Validating accuracy requires some other measure of the event collected at the same time or the ability to manually review the raw data (e.g., video for computer vision systems). Precision would be a measure of the consistency of the reported event or bout when determined multiple times under similar conditions. Digital data is particularly amenable to determining precision since the raw data is often archived and can be processed through the algorithm repeatedly to assess precision.

An additional consideration for algorithms that might be applied to raw data generated from more than one technology or varying experimental conditions (e.g., species, strain, cage environment conditions, etc.) is generalizability. Generalizability is a key issue for AI-defined algorithms that are ‘trained’ with examples of the behaviors or physiological events of interest. Generalizability refers to the varying spectrum of conditions under which the raw technology data is collected for which the AI algorithm performance would be expected to be consistent. Animals and cage conditions vary considerably across studies where animals’ color, size, and temperament vary; cage bedding varies, cage “furniture” and/or enrichment is variable, lighting conditions are different, sensor technologies may vary, etc. Generalizability explores how the algorithm performance varies across data derived from different sensor technologies or within the context of varying environmental cage conditions. The latter aspect is one of the weaknesses of deep learning based automated behavior recognizers: they perform very well in the context for which they have been trained but transfer poorly to other contexts (van Dam et al., 2020). A highly generalizable algorithm would maintain its performance across these varying conditions but that depends on the variety of conditions represented in the data used to train the algorithm and the specific feature being monitored. Accordingly, generalizability can be improved by training an algorithm with raw data collected under a broad spectrum of conditions. Generalizability may come at the expense of accuracy, i.e., reliability compared to the human observer (van Dam et al., 2020). This challenge can be overcome by training data from different contexts (van Dam et al., 2013).

Training an algorithm to handle every possible change in raw data isn't practical, so it would be beneficial for users to have a simplified validation process. This process would ensure that the algorithm is working correctly in their specific situations. This could be done using an internal control, which is a known data set that the algorithm has been tested on before, the use of orthogonal validation to cross-check the results, and/or a manual check of a small portion of the data to confirm the algorithm’s performance.

Analytical validation works best when AI scientists who created the algorithm work together with biologists who identified the biological event of interest and might have provided the data used to train the algorithm. A crucial part of this teamwork is defining the event, behavior, or physiology that the algorithm is detecting. For example, defining a mouse’s in-cage ‘locomotion’ derived from digital sensors can be more complex than cage-side observers agreeing the mouse is moving. One way to define locomotor activity could be a mouse moving more than 2 cm across a cage floor for at least 2 s, followed by at least 2 s of no movement with no maximum limit for these values. Alternatively, as in Penhold et al.'s study, where the activity of single-housed mice using technologies placed under the cage bottom that record changes in capacitance every 250 ms was assessed, activity or motion was defined as a change in Euclidean distance of ≥1 mm between samples (Pernold et al., 2023). Previous experiences were used to define local movement or movement-on-the-spot as being less than one average stride length and locomotion as when the distance covered at least one full stride length. Having a clear definition supports the validation process and lets the end-user know what biological endpoint is being measured. This clear definition ensures that a bout defined by the algorithm can be validated against a bout of similar definition, using any validation method.

As we’ve discussed above, many of today’s technologies are more precise than the human eye in identifying the details of a behavior. They can pinpoint the start and end of a behavior or event with great accuracy and likely greater precision. Accordingly, developing and improving algorithms is an iterative process, starting by defining the behavior based on direct observation, and then using the detailed data from the technologies to refine that definition based on experiential observations.

### 3.4 Clinical validation

Clinical validation follows analytical validation, where the primary aim of clinical validation is to support the clinical relevance of a digital measure that has demonstrated a sufficient level of analytical performance (e.g., sensitivity, specificity). Clinical validation is context-of-use (COU) dependent and is a process that confirms if a digital measure, like an algorithm’s output, accurately represents an animal’s health or disease status and helps to ensure the measure is biologically relevant. For example, clinical validation may provide confidence that measures of locomotor activity in a rat or mouse toxicology study are relevant for assessing drug-induced central (CNS) or peripheral nervous system (PNS) or musculoskeletal effects in that animal or that a measure of scratch reports the sensation of itch or the severity of cutaneous eosinophilic inflammation in a model of atopic dermatitis.

It is possible for a digital measure to be biologically or clinically relevant but lack the sensitivity to be clinically useful (e.g., inability to detect the onset of disease early enough to assess the ability to modulate the disease). The relevance of that measure in animals to the human condition is a different process of translational validation involving both the biological measure being collected but also the model in which it is being measured, which is outside the scope of this paper.

Clinical validation studies should be designed to represent the expected COU for a novel digital measure. Those studies should include not only the analytically validated digital measure but also an assessment that links the digital measure to the biology or pathobiology of interest. They may also include a traditional measure to demonstrate improved clinical usefulness. For example, a clinical validation study using the House Dust Mite mouse model that applies a digital measure of scratch might also include skin histopathology to correlate measures of scratch with the severity of eosinophilic inflammation that causes the itch that instigates scratching. Ideally, changes in measures of scratch would correlate with changes in the severity of inflammation. Likewise, that study could also include intermittent observational measures of scratch to demonstrate the usefulness of the more continuous and automated measures provided by digital technologies.

Clinical validation considers the biology represented by the quantitative digital measure and its relevance to the biology or pathobiology that the animal is being used to model. Importantly, the relevance of the measure may be reflected more by its change than its absolute quantity. For example, a measure of locomotor activity in a mouse may be within its physiological dynamic range but an increase or decrease in the presence of a drug may reflect the onset, progression, and resolution of a disease or toxicity. Alternatively, loss-of-righting reflex (LORR) is not generally considered a normal behavior so its detection may reflect a pathologic event. Likewise, its frequency and duration may represent the severity and progression of the CNS disease or injuries that caused it.

Though we often consider ‘clinical validation’ in the context of the relevance of our models and measures to our human clinical interests, these measures also often have relevance and usefulness in our efforts to optimally manage the welfare of our animal research subjects. Accordingly, many of these measures may also be used to identify animal health issues allowing us to mitigate pain and discomfort in those animals aside from the experimental intent.

Clinical validation is best performed by biologists who are familiar with the intended COU, who have experience in applying the models that define that context, and who understand the biology or pathobiology represented by the digital measure. A clinical validation could be one well-designed study that includes correlative endpoints, or a set of parallel studies conducted under similar conditions if technical incompatibilities prevent combining them in the same study. It could also be a series of studies that represent the variability in ways that the model or measure may be used. For example, EEG recordings may be a useful correlative endpoint that validates the association of LORR to a seizure event but might not be technically amenable to integrating into a computer vision-based approach to detecting loss of righting. Accordingly, parallel studies with those complementary endpoints could be run replicating study design elements as closely as possible.

## 4 Stakeholders and their roles and responsibilities with the V3 framework

Advancing the application of digital measures can be complex as it involves multiple stakeholders (Baran et al., 2022). This includes researchers whose primary interest is the collection of digital measures for scientific aims, animal care staff whose interests related to animal care and management, technology experts who oversee data storage and cybersecurity, data scientists who analyze the data, project teams that use study outcomes to inform decisions, business managers or operational managers who must approve purchase decisions, and finally, regulators who may use the results of studies to inform approvals. It is necessary to break down silos and foster collaboration to accelerate the application of these technologies, hence the development of the 3Rs Collaborative Translational Digital Biomarkers Initiative (www.3rc.org/tdb).

### 4.1 Role of collaboration and education

Interdisciplinary collaboration, sharing, and education are essential to progress the regulatory acceptance of digital measures with each stakeholder playing a unique role. Technology developers should provide data-driven presentations about their technologies and technical expertise to end-users and regulators. This is essential to introduce potential new users to these technologies and promote meaningful dialogue. End-users should, when possible, openly share case studies of successful regulatory submissions with digital measures data to multiply their efforts. This dissemination can be achieved through open-access presentations, publications, and even databases. Ideally, a database with easily understandable metadata and key sets related to regulatory submissions could be established to maintain confidential data, while allowing stakeholders to identify how digital measures are being used in different therapeutic fields or types of submissions. Of course, this can be a challenge with proprietary information. In turn, regulators can actively engage with digital measures in development, encourage submissions with digital measures, and communicate any specific requirements for digital measures submissions after they are developed.

Throughout the efforts to advance regulatory acceptance, it is essential to advance awareness of any developed frameworks through communication and training to relevant stakeholders about evaluation processes, methods, and findings. This ensures that stakeholders are aware of how various data and processes will be evaluated by regulators, while simultaneously encouraging the use of digital measures. Training is important to equip personnel with the skills to use the evaluation framework and navigate regulatory submissions using digital measure data. Proactive communication facilitates informed decision-making between all stakeholders.

## 5 Conclusion

In our pursuit to establish a robust evaluation framework for preclinical digital measures, we have chosen to adopt DiMe’s V3 framework as a foundational reference. By extrapolating the principles of this framework to the preclinical setting, we aim to foster a comprehensive and scientifically rigorous approach to digital assessments at this crucial phase of research. To provide clarity for all stakeholders, including end-users and regulators intending to adopt this approach, the framework is built on three cardinal pillars. The first, technology verification, emphasizes the necessity for devices to measure and store data with utmost precision, reliability, and reproducibility. The second, analytical validation, delves into the evaluation of data processing algorithms that transform raw technological readings into interpretable and actionable output metrics. The third, clinical validation, albeit adapted to a preclinical context, ensures that the digital tools effectively identify, measure, or predict relevant biological or functional states specific to the animal cohort and the defined context of use.

The *in vivo* V3 Framework provides a distinct advantage over the original DiMe V3 Framework for preclinical applications by tailoring its verification, analytical validation, and biological validation processes to accommodate the variability and complexity inherent in animal studies. By adapting these processes, the framework ensures that digital measures are validated in a way that reflects their translational relevance, thereby bridging the gap between preclinical findings and human clinical outcomes. This adaptation is crucial for enhancing the reliability and applicability of digital measures in drug development, ultimately supporting the transition from preclinical research to clinical trials. The *in vivo* V3 Framework’s emphasis on tailored validation, including rigorous sensor verification and replicability across species, makes it particularly suitable for the diverse and variable settings encountered in animal research. By elucidating each aspect of this *in vivo* V3 Framework for Preclinical Applications, we aim to equip stakeholders with a structured pathway to seamlessly integrate and validate digital measures within preclinical investigations.
